# Chitosan-Based Nanomaterials as Valuable Sources of Anti-Leishmanial Agents: A Systematic Review

**DOI:** 10.3390/nano11030689

**Published:** 2021-03-10

**Authors:** Hamdan I. AlMohammed, Amal Khudair Khalaf, Aishah E. Albalawi, Abdullah D. Alanazi, Parastoo Baharvand, Ali Moghaddam, Hossein Mahmoudvand

**Affiliations:** 1 Department of Microbiology and Parasitology, Almaarefa University, Riyadh 11597, Saudi Arabia; hamohammedii@mcst.edu.sa; 2 Department of Microbiology, College of Medicine, University of Thiqar, Thiqar 0096442, Iraq; amalkhudair111@yahoo.com; 3 Faculty of Science, University of Tabuk, Tabuk 47912, Saudi Arabia; ae.Albalawi@ut.edu.sa; 4 Department of Biological Science, Faculty of Science and Humanities, Shaqra University, P.O. Box 1040, Ad-Dawadimi 11911, Saudi Arabia; aalanazi@su.edu.sa; 5 Department of Medical Laboratory, Alghad International Colleges for Applied Medical Science, Tabuk 47913, Saudi Arabia; 6 Department of Social Medicine, School of Medicine, Lorestan University of Medical Sciences, Khorramabad 6813833946, Iran; dr.baharvand@gmail.com; 7 Student Research Committee, Lorestan University of Medical Sciences, Khorramabad 6813833946, Iran; alimogh7878@gmail.com; 8 Nutritional Health Research Center, Lorestan University of Medical Sciences, Khorramabad 6813833946, Iran

**Keywords:** cutaneous leishmaniasis, visceral leishmaniasis, promastigote, amastigote, alternative medicine, natural product

## Abstract

Background: The current chemotherapy agents against various forms of leishmaniasis have some problems and side effects, including high toxicity, high cost, and the emergence of resistant strains. Here, we aimed to review the preclinical studies (in vitro and in vivo) on the anti-leishmanial activity of chitosan and chitosan-based particles against *Leishmania* spp. Methods: This study was conducted based on the 06-PRISMA guidelines and registered in the CAMARADES-NC3Rs Preclinical Systematic Review and Meta-Analysis Facility (SyRF) database. Various English databases such as PubMed, Google Scholar, Web of Science, EBSCO, ScienceDirect, and Scopus were used to find the publications related to the anti-leishmanial effects of chitosan and its derivatives and other pharmaceutical formulations, without a date limitation, to find all the published articles. The keywords included “chitosan”, “chitosan nanoparticles”, “anti-leishmanial”, “*Leishmania*”, “leishmaniasis”, “cutaneous leishmaniasis”, “visceral leishmaniasis”, “in vitro”, and “in vivo”. The language for data collection were limited to English. Results: Of 2669 papers, 25 papers, including 7 in vitro (28.0%), 7 in vivo (28.0%), and 11 in vitro/in vivo (44.0%) studies conducted up to 2020 met the inclusion criteria for discussion in this systematic review. The most common species of *Leishmania* used in these studies were *L. major* (12, 48.0%), *L. donovani* (7, 28.0%), and *L. amazonensis* (4, 16.80%). In vivo, the most used animals were BALB/c mice (11, 61.1%) followed by hamsters (6, 33.3%) and Wistar rats (1, 5.5%), respectively. In vitro, the most used *Leishmania* form was amastigote (8, 44.4%), followed by promastigote (4, 22.2%), and both forms promastigote/amastigote (6, 33.3%). Conclusion: According to the literature, different types of drugs based on chitosan and their derivatives demonstrated considerable in vitro and in vivo anti-leishmanial activity against various *Leishmania* spp. Based on the findings of this review study, chitosan and its derivatives could be considered as an alternative and complementary source of valuable components against leishmaniasis with a high safety index. Nevertheless, more investigations are required to elaborate on this result, mainly in clinical settings.

## 1. Introduction

Leishmaniasis is a tropical and subtropical poverty-related disease caused by an intracellular parasite belonging to the genus *Leishmania* [1]. Humans are generally infected via the bite of a sandfly, mostly *Phlebotomus* and *Lutzomyia*, around the world [2]. According to reliable reports, 0.7–1 million new cases of the disease are notified annually, and 12–15 million people are now infected with the disease in different parts of the world [3,4]. Depending on the geographical distribution, various species of *Leishmania* such as *L. tropica, L. major*, *L. donovani*, *L. infantum*, *L. mexicana*, *L. braziliensis*, and *L. amazonensis* can cause different clinical forms of the disease [5,6]. Considering the classification and clinical picture of leishmaniasis in humans, the diseases are divided into four forms of cutaneous (CL), mucocutaneous (NCL), diffuse cutaneous (DCL), and visceral or kala-azar leishmaniasis (VL) [7,8].

There are a number of systemic and local therapeutic strategies for the treatment of various forms of leishmaniasis, including drugs (e.g., pentavalent antimony derivatives such as meglumine antimoniate (Glucantime^®^) and sodium stibogluconate (Pentostam^®^), miltefosine, pentamidine, amphotericin B (Amp B), and paromomycin), as well as physical treatments (e.g., cryotherapy, surgery, thermotherapy, and laser therapy) [9]. Based on recent studies, the current conventional chemotherapeutics generally have difficulty reaching the target tissues at the applied doses and are also linked to adverse side effects on healthy tissues [10], indicating that the drug delivery systems must improve the efficacy, tolerability, specificity, and therapeutic index of anti-leishmanial drugs. Moreover, unresponsiveness to these anti-leishmanial compounds, even to their higher doses, is regularly reported in some parts of the world [10,11]. These limitations motivate researchers to discover an effective alternative agent with low toxicity in natural compounds as a major source of medications with various therapeutics characteristics.

**Figure 1:**
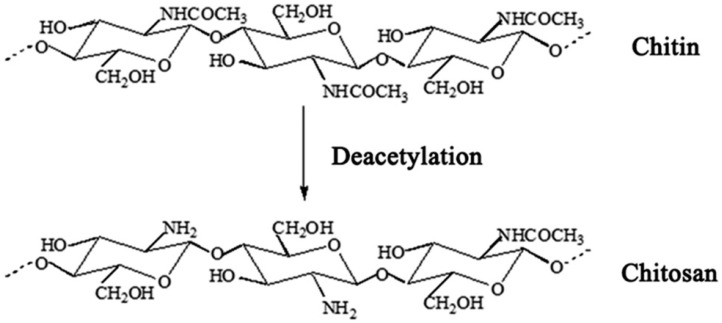
The chemical structure of chitin and chitosan.

Chitosan (poly-(b-1/4)-2-amino-2-deoxy-D-glucopyranose) is the general name used for a group of natural polysaccharide polymers produced by deacetylation of chitin (Figure 1) [12,13,14]. In recent years, the use of chitosan and its derivatives has attracted the attention of many researchers in medical and pharmaceutical sciences [15] due to its unique properties such as potent biological properties, low toxicity, biocompatibility, biodegradability, immunomodulatory [16], and anti-cancer, anti-nociceptive, anti-oxidant, anti-inflammatory, and anti-microbial properties [17,18].

Recent studies have demonstrated that the preparation of chitosan-based biomedical drugs such as nanoparticles, hydrogels, coatings, suspensions, powders, membranes, and films can impact the pharmaceutical and biomedical effects of these agents [19,20]. Recently, the antimicrobial activities of chitosan and its derivatives have been reported against a wide range of pathogenic viruses, bacteria, filamentous and yeast-like fungi [21,22], and helminthic and protozoan parasites [23,24]. Considering the anti-parasitic properties of chitosan and its derivatives, several investigations have demonstrated their potent anti-parasitic effects against some pathogenic strains such as *Cryptosporidium* spp. [25], *Echinococcus granulosus* [26], *Leishmania* spp. [23,24], and *Toxoplasma gondii* [27,28]. The present study aimed to review the preclinical studies (in vitro and in vivo) on the anti-leishmanial activity of chitosan and chitosan-based particles against *Leishmania* spp.

## 2. Methods

### 2.1. Database Search

This investigation was performed based on the 06-PRISMA guideline and registered in the CAMARADES-NC3Rs Preclinical Systematic Review and Meta-Analysis Facility (SyRF) database [29]. English databases, including PubMed, Google Scholar, Web of Science, EBSCO, ScienceDirect, and Scopus, were searched for publications related to anti-leishmanial effects of chitosan and its derivatives and other pharmaceutical formulations without a date limitation to identify all published articles. The keywords included “chitosan”, “chitosan nanoparticles”, “anti-leishmanial”, “*Leishmania*”, “leishmaniasis”, “cutaneous leishmaniasis”, “visceral leishmaniasis”, “in vitro”, and “in vivo”. Moreover, the language for data collection was limited to English.

### 2.2. Quality Assessment and Article Selection

The studies evaluating the anti-leishmanial effects of chitosan and its derivatives were examined. First, the studies were imported into EndNote X9 (Thomson Reuters, New York, NY, USA) and the duplicates were removed. Afterwards, three authors independently examined the title and abstract of the studies, and the relevant documents about the in vitro and/or in vivo anti-leishmanial effects of chitosan, chitosan derivatives, and chitosan-based nanoparticles were included for further analysis. The same authors carefully read the studies and selected the eligible investigations that adequately met the inclusion criteria. The inclusion criteria were articles evaluating the anti-leishmanial effects of chitosan and its derivatives and emphasizing the design of various forms of nanoparticles containing chitosan and other pharmaceutical formulations against leishmaniasis.

A total of 2669 articles were identified through database searching. Among these articles, 227 articles were removed due to duplication. Of the remaining 2442 articles, 2385 studies were removed due to the inadequate information and the ones in which the abstract was submitted in congresses as preceding papers, conferences, and editorials without full text. One of the main limitations in such studies is the frame of differing access to full texts between different research locations of study, which prevents us from accessing the full-text of some articles. To solve this problem, we searched in various research websites such as ResearchGate and LinkedIn which suggest a choice of direct full-text request from authors as well as exploring archives of wanted journals, or contacting principal investigator to purchase it if available.

**Figure 2:**
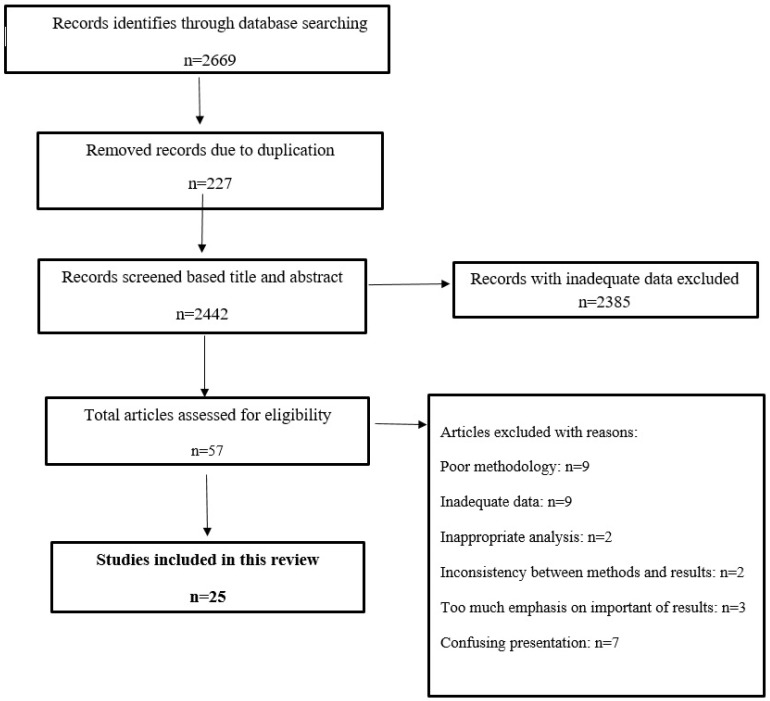
Flowchart describing the study design process.

From the remaining 57 articles which were assessed for eligibility, 32 articles were excluded due to some reasons such as inconsistency between methods with results, incorrect interpretation of the results, poor methodology, etc.; whereas 32 articles were finally included in this review (Figure 2).

### 2.3. Data Extraction

Three authors independently extracted information from the selected articles and, if necessary, the differences were resolved upon discussions with the corresponding author. The extracted data included chitosan type, whether it was used in combination or loaded with other drugs, the type of study, animals, doses, time, important results, and references.

## 3. Results and Discussion

Chitosan as a natural agent with diverse biological activities is generally found in the shells of crustaceans, such as crab, shrimp, squid pen, and crawfish; however, recent investigations have reported that chitosan can be produced from some fungi [15,16,17]. Of 2669 papers, 25 papers, including 7 in vitro (28.0%), 7 in vivo (28.0%), and 11 in vitro/in vivo (44.0%) studies conducted up to 2020 met the inclusion criteria for discussion in this systematic review. Totally, the most common species of *Leishmania* used in these studies were *L. major* (12%, 48.0%), *L. donovani* (7%, 28.0%), and *L. amazonensis* (4%, 16.80%), respectively.

### 3.1. Chitosan Treatments In Vitro

In vitro, the most used *Leishmania* form was amastigotes (8%, 44.4%), followed by promastigotes (4%, 22.2%), and both forms promastigotes/amastigotes (6%, 33.3%). In terms of the concentrations of chitosan and its formulations, the results demonstrated that, *in vitro*, they were used in the range of 0.03 µg/mL to 20 mg/mL. The findings demonstrated that the most used synthetic drugs for combination therapy in vitro were amphotericin B (14%, 70.0%), followed by miltefosine (3%, 15.0%) and doxorubicin hydrochloride (2%, 10.0%). The most common species of *Leishmania* used in in vitro studies were *L. major* (8%, 44.4%), *L. donovani* (6%, 33.3%), and *L. amazonensis* (3%, 16.6%), respectively (Table 1).

The results exhibited that the most used in vitro screening strategy to check the anti-leishmanial effects of chitosan and its derivatives were the intracellular living amastigote assay, followed by the extracellular promastigote assay. Now, various in vitro systems are used in the evaluation of agents, which show direct toxic action on the parasite [48]; however, for some agents that act through their metabolites and/or host defense system, it does not seem like a good option [49]. Some in vitro screening studies target the promastigote forms of *Leishmania* because of the simplicity of the cultivation and handling of parasites, the short time required and the low cost [50]; nevertheless, because of some weaknesses, for example, the lack of host cells in this method and the presence of this parasitic form in the invertebrate vector, it is not considered a reliable target to test anti-leishmanial compounds [51]. The use of intracellular amastigote forms in the in vitro system is also well-known as a more clinically relevant model for the assessment of anti-leishmanial compounds due to having some unique features such as acquiring important cell-health information, low cost, and no need for secondary assays [52,53].

Previously, Mohebali et al. [31] have reported that the chitosan at the doses of 100, 200 and 400 μg/mL completely killed the *L. major* promastigotes in vitro. In another in vitro study conducted by Feizabadi et al. [37], the results showed that chitosan coupled with *L. major* secretory and excretory proteins can increase the ability of infected macrophages to remove parasites by reducing apoptosis. Riberio et al. (2014) have reported that chitosan nanoparticles combined with AmpB significantly reduced the lesion size and parasite burden in all the evaluated organs of mice infected with *L. amazonensis*. They concluded that this compound controls leishmaniasis by increasing the cytokines of IFN-γ, IL-12, IL-4, and IL-10 in the infected mice [54].

### 3.2. Chitosan Treatments In Vivo

The obtained results showed that the most used animals in vivo were BALB/C mice (11%, 61.1%) followed by hamsters (6%, 33.3%) and Wistar rats (1%, 5.5%). In terms of the concentrations of chitosan and its formulations, the results demonstrated that, in vivo, they were used in the range of 0.005 mg/kg to 500 mg/kg. The most common species of *Leishmania* used in in vitro studies were *L. major* (9%, 44.4%), followed by *L. donovani* (7%, 33.3%) (Table 2).

In vivo assay or using the standardized animal models in laboratories are considered as the best strategies to evaluate the anti-leishmanial drug compounds against various forms of leishmaniasis which has the closest resemblance to the human condition [61]. Although the predictive power of the in vivo assay is not very noticeable, however, the important points in this model are that (i) if a drug is not effective in vivo, there is no need to study further and that (ii) some useful information is obtained about the effective doses as well as the toxicity of the studied drugs [48]. Today, several in vivo experimental models are used to assess and test novel agents against various forms of leishmaniasis, e.g., (i) the BALB/C mice-*L. major* model considered as a validated model for human CL; (ii) C57BL/6, BALB/C, and CBA/J mice to induce *L. amazonensis*, *L. mexicana* infection; (iii) BALB/C mice and Syrian golden hamster as experimental models for *L. infantum* infection [48,49].

Mohebali et al. [30] have demonstrated that the chitosan at the doses of 100, 200 and 400 μg/mL reduced the size of the lesion from 10.7 ± 3.24 mm in the control group to 1.05 ± 1.02 mm on day 28 at a dose of 400 μg/mL, and also from 6.27 ± 1.23 mm to 2.07 ± 0.87 mm in the 200 μg/mL concentration in the mice infected with *L. major.* Mohammadi-Samani et al. [56] showed that superoxide dismutase B1 combined with chitosan nanoparticles is able to promote the immunogenicity to cell-mediated immunity (T(H)1 cells producing IgG2a in mice) that is effective in removing of *Leishmania* parasites and might be considered as a single-dose nanovaccine for leishmaniasis.

### 3.3. Treatments Using Chitosan as Vehicle

Nowadays, it has been demonstrated that chitosan, its derivatives, and chitosan-based nanomaterials are able to possibly remove barriers in the carrying of drugs, thus improving the efficacy of the drug and subsequently the targeted drug therapy [62]. The findings demonstrated that the most used synthetic drugs for combination therapy in vivo and in vitro were amphotericin B (14%, 70.0%), followed by miltefosine (3%, 15.0%) and doxorubicin hydrochloride (2%, 10.0%). Although most of the studies in this review use chitosan in combination with other drugs, however, chitosan and its derivatives without combination with common drugs have been considered in some studies.

Malli et al. (2019) have also demonstrated that the nanoparticles of poly (isobutyl-cyanoacrylate) coated with chitosan have potent anti-leishmanial effects on *L. major* promastigotes through morphological changes such as the aberrant shape and swelling of mitochondria and parasitic vacuoles [45]. In a study conducted by Feizabadi et al. (2019), it has been proven that chitosan combined with *L. major* secretory and excretory proteins can improve the ability of infected macrophages to remove parasites by decreasing apoptosis [37].

For example, Lima et al. [32] have reported that chitosan-silver nanoparticles have more anti-leishmanial activity than chitosan on *L. amazonensis* promastigotes with the IC_50_ values of 1.69 and 7.81 µg/mL, respectively. In the study conducted by Seyyed Tabaei et al. [44] have showed that chitosan-polyethylene oxide-berberine nanofibers has potent therapeutic effects on healing of CL induced by *L. major* in BALAB/C mice through reducing the parasite burden, decreasing the lesion size as well as change in the epidermis and dermis.

In recent years, the anti-parasitic activities of chitosan and its various derivatives/formulations have been studied against several parasitic pathogens such as *C. pavum* [25], *Echinococcus* spp. [26], and *T. gondii* [27,28]. For example, Mammeri et al. (2018) demonstrated that chitosan significantly decreased the viability of *Cryptosporidium parvum* oocysts by >95% after 24 h of treatment with chitosan mix (C-Mix) and chitosan N-acetyl-D-glucosamine (CNAD). They also reported that C-Mix (34.5%) and CNAD (56%) significantly decreased the oocysts’ shedding by 34.5% and 56% in newborn mice infected with cryptosporidiosis, respectively [25]. Torabi et al. (2018) have demonstrated that chitosan-praziquantel and chitosan-albendazole nanoparticles especially in combination at the doses of 1, 5, and 10 μg/mL significantly reduced the viability of microcysts, weight and number of cysts in vitro and in vivo [26]. In the study conducted by Teimouri et al. (2018), it has been proven that low molecular weight chitosan nanoparticles completely killed the tachyzoites at the concentration of 500 and 1000 ppm in vitro; they also showed that this compound considerably increased the survival time of infected mice with *T. gondii* RH strain from 6 to 8 days after infection [27].

### 3.4. Possible Antimicrobial Mechanisms of Chitosan

The precise antimicrobial mechanism of action of chitosan is yet to be fully understood; still, based on the literature, the most likely antimicrobial mechanisms of action of chitosan include the disruption of the cell wall and, consequently, an effect on the membrane’s permeability, inhibition of DNA replication, cell death, and bindings to the trace metal elements resulting in toxin production and microbial growth inhibition [63].

Mohammadi-Samani et al. (2011) have reported that chitosan nanoparticles containing *Leishmania* superoxide dismutase could be considered a nano-vaccine for leishmaniasis eradication by promoting the immune response toward cell-mediated immunity (TH1 cells producing IgG2a in mice) [56].

### 3.5. Cytotoxicity Effects of Chitosan

With respect to the cytotoxic effects of chitosan and its various formulations, Karam et al. (2020) found that chitosan nanocapsules containing the essential oil of *Matricaria chamomilla* have no significant cytotoxicity against macrophage cells with a CC_50_ (the 50% cytotoxic concentration) value of 207.92 ± 18.53 μg/mL compared to 19.71 ± 1.73 μg/mL for essential oil alone [35]. Another study conducted by Chaubey et al. (2018) indicated that the mannose-conjugated chitosan nanoparticles of curcumin had no significant cytotoxicity against the J774A.1 macrophage cell line with a CC_50_ value of 26 ± 0.60 mg/mL [41]. Recently, Esfandiari et al. (2019) have reported that paromomycin-loaded mannosylated chitosan nanoparticles had no considerable cytotoxicity against the human monocyte cell line of THP-1 cells with a CC_50_ value of 3911 μg/mL [43].

## 4. Conclusions

Studies in recent years revealed that chitosan, its derivatives, and chitosan-based nanomaterials are able possibly remove barriers in the carrying of drugs thus improving the efficacy of the drug and subsequently the targeted drug therapy. In the present review, based on the literature, various forms of drugs based on chitosan and their derivatives exhibited significant antileishmanial activity against various *Leishmania* spp, in vitro and in vivo. The results showed that chitosan and chitosan-based particles could be considered as an alternative and complementary source of valuable components against leishmaniasis with a high safety index. However, more studies are required to elucidate this finding, particularly in clinical settings.

## Figures and Tables

**Table 1:** A list of in vitro studies of chitosan and its derivatives as resource for anti-leishmanial agents.

Type of Chitosan	Combined with the Drug	Year of Publication	Parasite Form	*Leishmania* spp.	Concentration	Time	Outcome	Ref.
AmB-loaded pluronic F127 (PF 127) micelles coated with chitosan (Cs-PF-AmB-M)	Amphotericin B (AmpB)	2017	Promastigotes	*Leishmania donovani*	0.03, 0.05, 0.1, 0.2, 0.4, 0.8 µg/mL	72 h	Experiments have shown that Cs-PF-AmB-M at a dose of 0.049 µg/mL may reduce parasitic load by 50%; whereas PF-AmB-M at a dose of 0.08 µg/mL reduced parasitic load by 50%.	[30]
Chitosan	-	2018	Promastigotes	*L. major*	50, 100, 200, 400 μg/mL	30, 60, 120 and 180 min	The results showed that chitosan at the concentrations of 200 and 400 μg/mL after 180 min killed 100% of promastigote.	[31]
Chitosan-based silver nanoparticles	-	2017	Promastigotes and amastigotes	*L. amazonensis*	0.42 to 27µg	48 h	The results showed that this compound has potent anti-leishmanial effects against promastigote and amastigote stages of *L. amazonensis* after 48 h exposure, with IC_50_ values ranging from 0.422 to 2120 μg/mL.	[32]
Chitosan microparticles	Doxorubicin hydrochloride (DOX)	2011	Promastigotes	*L. donovani*	0.03, 0.08, 0.13 and 0.2 mg/mL	20 h	The results showed that the greatest effect of these microparticles was in the first 60 min and caused nonspecific activation of phagocytosis in macrophages.	[33]
Chitosan anchored nanostructured lipid carriers (NLC)	Miltefosine (HePC- hexadecyl phosphocholine) and amphotericin B (AmB)	2017	Amastigotes	*L. donovani*	50, 100, 250, 500, 1000 ng/mL	4 h	The results showed that the highest effect of these nanoparticles was at a concentration of 1000 ng/mL, which killed more than 80% of amastigotes, while AmB alone reduced the parasitic load by about 60%.	[34]
Chitosan nanocapsules containing essential oil of *Matricaria chamomilla* *(NCEO)*	-	2020	Promastigotes	*L. amazonensis*	0.1–1000 μg/mL	48 h	The results showed that IC_50_ NCEO was 7.18 ± 0.7 μg/mL against promastigotes and 14.29 ± 1.01 μg/mL against amastigotes.	[35]
Chitosan nanoparticles (CNPs) and, 4-SO4GalNAc modified chitosan nanoparticles (SCNP)	Amphotericin B (AmpB)	2015	Amastigotes	*L. donovani*	0.05, 0.1, 0.2. 0.4. 0.8 (µg/mL)	24 h	The results showed that AmB-SCNPs and AmB–CNPs had a better effect in comparison with amphotericin B and more than 80% of their lethality was recorded, while for amphotericin B 70% lethality have been recorded.	[36]
Chitosan nanoparticles	-	2019	Amastigotes	*L. major*	5–250 µL/mL	48 h	Chitosan coupled with *L. major* secretory and excretory proteins can increase the ability of infected macrophages to remove parasites by reducing apoptosis.	[37]
Chitosan nanoparticles	Miltefosine	2020	Promastigotes and amastigotes	*L. tropica*	100 µL/mL	72 h	The results showed that IC_50_ value for promastigote and amastigote forms of *L. tropica* was 0.07 ± 0.05 µL/mL and 0.09 ± 0.02 µL/mL, respectively,	[38]
Chitosan nanoparticles and sodium tripolyphosphate (TPP)	Amphotericin B (AmB)	2020	Amastigotes	*L. major* and *L. mexicana*	1 mg/mL	7 days	The results showed that EC_50_ value of AmB-CH-TPP for *L. major and L. mexicana* amastigotes was 0.14 ± 0.09 µg/mL and 0.5 ± 0.01 µg/mL, respectively.	[39]
Chitosan-polyethylene oxide nanofibers containing berberine	-	2020	Promastigotes and amastigote	*L. major*	0.01–50 μg/mL	24, 48, 72 h	The results showed that this compound has potent anti-leishmanial effects against promastigotes and amastigotes of *L. major* with IC_50_ values ranging from 0.197 to 1.023 μg/mL.	[40]
Curcumin-loaded mannose-functionalized chitosan nanoparticles (Cur-MCN)	-	2018	Amastigotes	*L. donovani*	0.05–2.0 mg/L	72 h	The results showed that Cur-MCN at the concentration of 0.518 ± 0.01 mg/L reduced 50% of amastigotes; also, no toxic effect on macrophages was observed in the use of Cur-MCN.	[41]
Encapsulate S-nitroso-mercaptosuccinic acid into chitosan nanoparticles (NONPs)	-	2019	Promastigotes and amastigotes	*L. amazonensis*	25, 50, 75, 100, 200, 400 µM	24 h	Experiments on amastigotes and promastigotes of *L. amazonensis* showed that NONPs reduced 65% of the parasitic load at a dose of 200 µM and killed 85% of promastigotes at a dose of 75 µM. These nanoparticles also reduced the number of amastigotes from 8.5 ± 1.2 in the control group to 4.5 ± 0.4 per 300 macrophages and reduced the infection rate from 76.2 ± 7.1 to 63.7 ± 5.4.	[42]
Mannosylated chitosan (MCS) with dextran (dex)	Paromomycin (PM)	2019	Amastigotes	*L. major*	5, 10, 20, 40, 80, 160, 320 μg/mL	24, 48 h	The results showed that this compound has no cytotoxicity on macrophages and at a dose of 5 μg/mL reduced more than 60% of the parasitic load inside macrophages.	[43]
Nanosized chitosan-betulinic acid	-	2020	Promastigotes and amastigote	*L. major*	20 μg/mL	48 h	The results showed that BK20 (20 μg/mL) was effective to kill the parasite by 86% compared to negative control group. The infection rate and the mean number of amastigotes per each macrophage were found to be 73% and 7%, respectively.	[44]
Oleoyl chitosan and α-cyclodextrin (α-CD)	-	2019	Amastigotes	*L. major*	100 μL	4 days	The results showed that the use of oleoyl chitosan/α-CD platelets at a dose of 60.24 ± 4.42 μg/mL killed 50% of amastigotes.	[45]
Poly (isobutylcyano acrylate) nanoparticles coated with chitosan (Cs-NPs)	Amphotericin B-deoxycholate (AmB-DOC)	2019	Promastigotes and amastigote	*L. major*	20 mg/mL	10 min, 20 min, 30 min, 1 h, or 2 h	The IC_50_ values for *L. major* promastigote and axenic amastigote forms were 1.14 ± 0.11 μg/mL and 0.53 ± 0.07 μg/mL, respectively.	[46]
Sodium alginate-glycol chitosan stearate nanoparticles (SA-GCS-NP)	Amphotericin B (AmB)	2015	Amastigotes	*L. donovani*	10 ng/mL	48 h	The IC_50_ values of AmB-SAGCS-NP and AmB for amastigotes of *L. donovani* were 0.128 ± 0.024 μg/mL and 0.214 ± 0.06 μg/mL, respectively.	[47]

**Table 2:** A list of in vivo studies of chitosan and its derivatives as resources for anti-leishmanial agents.

Type of Chitosan	Combined with the Drug	Method	Administration	Animal	*Leishmania* spp.	Dose	Time	Outcome	Year of Publication	Ref.
AmB-loaded pluronic F127 (PF 127) micelles coated with chitosan (Cs-PF-AmB-M)	Amphotericin B (AmB)	Film hydration method	Intraperitoneal	Syrian golden hamster	*Leishmania donovani*	1 mg/kg	5 days	The results showed that Cs-PF-AmB-M and PF-AmB-M significantly reduced the parasite load; also, the number of amastigotes was significantly reduced by 52.67 ± 17.24.	2017	[30]
Chitosan	-	-	Topically	BALB/c mice	*Leishmania major*	200, 400 μg/mL	28 days	Chitosan reduced the size of the lesion from 10.7 ± 3.24 mm in the control group to 1.05 ± 1.02 mm on day 28 at a dose of 400 μg/mL.	2018	[31]
Chitosan microparticles	Doxorubicin hydrochloride (DOX)	-	Intraperitoneal	Golden hamsters	*Leishmania donovani*	500 mg/kg	7 days	The results showed that this compound killed 78.2 ± 10.4% of amastigotes.	2011	[33]
Chitosan anchored nanostructured lipid carriers (NLC)	Miltefosine (HePC- hexadecyl phosphocholine) and amphotericin B (AmB)	-	Intravenous	Naive hamsters	*Leishmania donovani*	1 mg/kg	5 days	The results showed that HePC-AmB-CNLCs could reduce the parasitic load by 88.14 ± 4.12%, while tween 80-AmB-CNLCs and AmB reduced the parasite load by 70.91 ± 3.5% and 53.26 ± 2.5%, respectively.	2017	[34]
Chitosan nanocapsule (CNC)	Amphotericin B	Emulsification n-solvent evaporation	Intraperitoneal	Syrian golden hamsters	*Leishmania donovani*	1 mg of drug/kg	30 days	The results showed that this compound killed 86.1 ± 2.08% of *Leishmania* amastigotes.	2013	[55]
Chitosan nanoparticles	-	Ionotropic gelation process	Subcutaneously	BALB/c mice	Leishmania major	5 μg/50 μL	3 weeks	The results showed that injection of this compound in BALB/c mice could activate TH1 cells and IgG2a and eradicate *Leishmania* with cell-mediated immunity.	2011	[56]
Chitosan nanoparticles	Amphotericin B (AmpB)	Polyelectrolyte complexes technique	Intravenous	BALB/c mice	*Leishmania amazonensis*	100 μL/kg	10 days	The results showed that the combined use of chitosan and chondroitin sulfate nanoparticles with amphotericin B can significantly reduce the lesion size and parasitic load and also provide higher levels of IFN-γ and IL-12.	2014	[54]
Chitosan nanoparticles (CNPs) and, 4-SO4GalNAc modified chitosan nanoparticles (SCNP)	Amphotericin B (AmpB)	Ionic gelation	Intravenous	Wistar rats	*Leishmania donovani*	1 mg/kg	0.5, 1, 2, 4, 6 and 24 h	The results showed that the use of AmB-SCNPs reduced the load of parasites in the spleen by 75.30 ± 3.76%, but the use of AmB-CNPs and amphotericin B alone kills 63.89 ± 3.44% and 47.56 ± 2.37% of parasites.	2015	[36]
Chitosan nanoparticles and sodium tripolyphosphate (TPP)	Amphotericin B (AmB)	Dextran sulphate aqueous solution	Intravenous	BALB/c mice	*Leishmania major*	1.25, 2.5, 5 mg/kg	10 days	AmB-CH-TPP at a dose of 5 mg/kg reduced the size of the lesion by 83% and also reduce the parasitic load by 99%, but CH-TPP only reduced 35% of the lesion size and 65% of parasitic load.	2020	[39]
Chitosan platelets	Amphotericin B-deoxycholate	-	Intralesional	BALB/c mice	*Leishmania major*	100 μL/kg	13 days	The results showed that the use of AmB-DOC and the chitosan platelets caused thickening and dry scales on the lesion, which indicated improvement; granuloma spread in these mice is more limited and the number of infected macrophages is less than the use of AmB-DOC.	2019	[45]
Curcumin-loaded mannose-functionalized chitosan nanoparticles (Cur-MCN)	-	-	Intraperitoneal	Hamster	*Leishmania donovani*	50 mg/kg	5 days	The results showed that Cur-MCN have more anti-leishmaniasis properties than curcumin alone and are also more efficient at drug delivery than Cur-CN (curcumin-loaded unconjugated chitosan nanoparticles). Cur-MCN were able to reduce the parasitic load in the spleen by 94.20% and the number of amastigotes from 1647 ± 125.2 in the control group to 112 ± 32.2 per 500 macrophages.	2018	[41]
Nano chitosan	Amphotericin B	-	Intralesional	BALB/c mice	*Leishmania major*	5, 7, 10 mg/kg	3 weeks	The results showed that this compound improved the lesion and reduce its diameter to 0 mm and killed 81% of amastigotes; additionally, no mortality was reported in mice after using this compound; while using amphotericin B alone, 10% of mice died, and no toxicity or side effects were reported.	2018	[57]
Poly (isobutylcyano acrylate) nanoparticles coated with chitosan (Cs-NPs)	Amphotericin B-deoxycholate (AmB-DOC)	-	Topically	BALB/c mice	*Leishmania major*	100 μL/kg	13 days	The results showed that topical application of this compound with or without AmB-DOC on the skin of *L. major* mice could cause a slight improvement of the CL lesion; also, the collected skin samples showed that this combination reduces the parasitic load.	2019	[46]
Sodium alginate-glycol chitosan stearate nanoparticles (SA-GCS-NP)	Amphotericin B (AmB)	Ionotropic complexation method	Intraperitoneally	Syrian golden hamsters	*Leishmania donovani*	5, 10, 20 mg/kg	5 days	The results showed that AmB-SAGCS-NP reduced 70.21 ± 3.46% of the parasitic load, while AmB kills only 53.24 ± 2.84% of amastigotes.	2015	[47]
β-lapachone (βLP) in lecithin-chitosan nanoparticles (NP)	-	-	Topically	BALB/c mice	*Leishmania major*	20 mg/kg	21 days	The use of these nanoparticles in CL reduced the number of amastigotes from 46 to 11 per 100 macrophages; also, these nanoparticles reduced the size of the lesion from 61.2 ± 21.2 mm^2^ to 35.7 ± 29.4 mm^2^.	2015	[58]
Nanosized chitosan-betulinic acid	-	Drug adsorption and phase separation	Intraperitoneally	BALB/c mice	*Leishmania major*	20 mg/kg	28 days	The lesion size in positive control group (GUL200) was negligibly decreased to 1.2 mm; also, in B20 mg/kg and K12.5 mg/kg receiver mice, the lesion size was slightly decreased, while in the group of BK20 mg/kg, the lesion size was considerably decreased and reached to zero (*p* < 0.001)	2018	[59]
Chitosan-based nano-scaffolds	-	Electrospinning method	Topically	BALB/c mice	*Leishmania major*	20 wt%	28 days	This compound significantly reduced skin ulcer diameter (*p* = 0.000), parasite burden (*p* = 0.003), changes in the epidermis (*p* = 0.023), and dermis (*p* = 0.032); indicated significantly strong effectiveness of the produced nano-scaffolds against *Leishmania* ulcers.	2020	[44]
N-Palmitoyl-N-monomethyl-N,N-dimethyl-N,N,N-trimethyl6-O-glycol chitosan nanoparticles (GCPQ)	Amphotericin B	-	Orally	BALB/c mice	*Leishmania* *infantum*	5 mg/kg	10 days	AmB-GCPQ nanoparticles demonstrated higher efficacy compared with parenteral liposomal AmB.	2015	[60]

## Data Availability

All data generated or analyzed during this study are included in this published article.
